# Size and Fiber Density Controlled Synthesis of Fibrous Nanosilica Spheres (KCC-1)

**DOI:** 10.1038/srep24888

**Published:** 2016-04-27

**Authors:** Nisha Bayal, Baljeet Singh, Rustam Singh, Vivek Polshettiwar

**Affiliations:** 1 Nanocatalysis Laboratories (NanoCat), Department of Chemical Sciences, Tata Institute of Fundamental Research (TIFR), Mumbai, India

## Abstract

We report a facile protocol for the synthesis of fibrous nano-silica (KCC-1) with controllable size and fiber density. In this work, we have shown that the particle size, fiber density, surface area and pore volume of KCC-1 can be effectively controlled and tuned by changing various reaction parameters, such as the concentrations of urea, CTAB, 1-pentanol, reaction time, temperature, solvent ratio, and even outside stirring time. For the first time, we were able to control the particle size ranging from as small as 170 nm to as large as 1120 nm. We were also able to control the fiber density from low to medium to very dense, which consequently allowed the tuning of the pore volume. We were able to achieve a pore volume of 2.18 cm^3^/g, which is the highest reported for such a fibrous material. Notably we were even able to increase the surface area up to 1244 m^2^/g, nearly double the previously reported surface area of KCC-1. Thus, one can now synthesize KCC-1 with various degrees of size, surface area, pore volume, and fiber density.

High surface area silica has wide applications in almost every field^1^,^2^,^3^,^4^,^5^. Importantly, the inherent properties of silica can be tuned by varying different parameters such as the size, shape, and morphology^6^,^7^,^8^,^9^,^10^,^11^,^12^. The demand for silica nanospheres with different sizes, dimensions and morphology is continuously growing because modern industries have spurred a tremendous interest for such materials^13^,^14^,^15^,^16^.The effectiveness of these materials is mainly due to their micro and mesostructures, which allow active molecules to disperse on the large internal surface, improving the activity. The accessibility of active sites inside the nano-silica particles is crucial, as poor accessibility will limit their applications when significant mass transport is vital. Therefore, a high surface nano-silica with better accessibility was needed.

We recently reported the synthesis of a new class of fibrous nano-silica (KCC-1)^17^,^18^,^19^,^20^,^21^,^22^,^23^,^24^,^25^,^26^,^27^. The fibrous morphology observed in these nanospheres has not been seen before in silica materials. KCC-1 exhibits excellent physical properties, including a high surface area, fibrous morphology, good thermal/hydrothermal properties, and high mechanical stability. The uniqueness of KCC-1 is its high surface area is by virtue of its fibrous structure instead of pores (unlike MCM-41 and SBA-15 silicas), making it easily accessible. We also showed the successful utilization of nano-silica (KCC-1) for a range of important applications^18^,^19^,^20^,^21^,^22^,^23^,^24^,^25^,^26^. Metal nanoparticles supported on KCC-1 showed a multifold increase in their catalytic activity and stability compared to MCM-41 and SBA-15-supported catalysts for various challenging reactions such as metathesis, hydrogenolysis, and C-C coupling reactions^18^,^19^,^20^. Tantalum hydride (TaH) supported on KCC-1 catalyzed a new hydro-metathesis reaction of olefins with remarkable catalytic activity and stability^21^. Along similar lines, KCC-1-based oxynitrides showed multifold enhancement in activity as a solid base compared to other known catalysts^22^,^23^. This enhancement in activity was explained on the basis of the excellent accessibility of the active sites due to the open and flexible fibrous structure of KCC-1^22^, as well as the different amine groups present on the surface^23^. KCC-1 oxynitrides as well as KCC-1-amines were also used to capture CO_2_^24^,^25^, and they showed good CO_2_ capture capacity, fast kinetics, easy regeneration, excellent mechanical strength, and high thermal stability. Very recently we reported the design and synthesis of high surface area photocatalysts by coating TiO_2_ on the fibrous nano-silica (KCC-1) using atomic layer deposition (ALD)^26^. Our developed catalyst showed extraordinary photo-catalytic activity, far better than well-known MCM-41 and SBA-15 supported TiO_2_ catalysts as well as other silica supported TiO_2_ catalysts reported in the literature. Notably, for the first time, we also observed formation of small and monodispersed TiO_2_ quantum dots after heat treatment of these catalysts^26^. Asefa *et al.* demonstrated the use of KCC-1 for its high DNA adsorption capacity and effective *in vitro* delivery of genes^27^. All in all, it is the fibrous morphology of KCC-1 that provides better accessibility of the active sites for enhanced catalytic activities and capture efficiencies.

After our discovery of KCC-1 in march 2010, several reputed groups worldwide reported the successful use of KCC-1 for various applications such as catalysis, photocatalysis, CO_2_ capture-conversion, sensing, detection and extraction of ions, supercapacitors, drug delivery and other biomedical applications^28^,^29^,^30^,^31^,^32^,^33^,^34^,^35^,^36^,^37^,^38^,^39^,^40^,^41^,^42^,^43^,^44^,^45^,^46^,^47^,^48^,^49^,^50^,^51^,^52^,^53^,^54^,^55^,^56^. Although KCC-1 nano-spheres possess unique textural and physical properties and show a dramatic enhancement in activity, control over their particle size, fiber density and textural properties (surface area, pore volume, pore size) has not been achieved yet^17^,^18^,^19^,^20^,^21^,^22^,^23^,^24^,^25^,^26^,^27^,^28^,^29^,^30^,^31^,^32^,^33^,^34^,^35^,^36^,^37^,^38^,^39^,^40^,^41^,^42^,^43^,^44^,^45^,^46^,^47^,^48^,^49^,^50^,^51^,^52^,^53^,^54^,^55^, despite their being critical parameters for the successful development of KCC-1-supported catalysts/photocatalysts and sorbents as well as in drug delivery. For example, drug delivery applications need smaller particles (smaller than 200 nm)^16^, while catalysts will be more stable if the particle size is larger (without compromising the surface area) due to the minimization of aggregation/sintering. Light-scattering events can be increased by using larger-sized KCC-1 particles with a higher surface area, which will directly result in better photocatalytic applications. The fiber density can also be used to harvest light by increasing its internal reflection/scattering by tuning its internal structure. Fiber density control will allow the tuning of the pore size (in this work, defined as the space between two fibers), which will allow the tuning of the selectivity in catalysis and adsorption applications.

Thus, there is an urgent need to develop a simple and sustainable protocol for the synthesis of monodisperse KCC-1 with controllable size, fiber density and textural properties. In a continuation of our research on KCC-1, in this work, we report a facile synthetic protocol to control the size, fiber density, surface area, pore volume and pore size distribution. The developed protocol is simple and exerts extraordinary control over the size, fibrous density and textural properties of KCC-1.

## Results and Discussion

The synthesis of KCC-1 was carried out by modification of our previously reported protocol^17^ based on the microwave (MW)-assisted solvo-thermal heating of tetraethyl orthosilicate (TEOS) using urea as a hydrolyzing/condensation reagent and cetyltrimethylammonium bromide (CTAB) as a template in a water: cyclohexane: 1-pentanol solvent mixture at 120 °C. Several synthetic parameters, such as the urea concentration, CTAB concentration, reaction time, reaction temperature, 1-pentanol concentration, solvent ratios and outside stirring time, were systematically studied for their effect on the size, fiber density and textural properties (surface area, pore volume and pore size) of KCC-1. The as-synthesized materials were named as KCC-1 (X), where X is the average particle size (APS).

**Figure 1:**
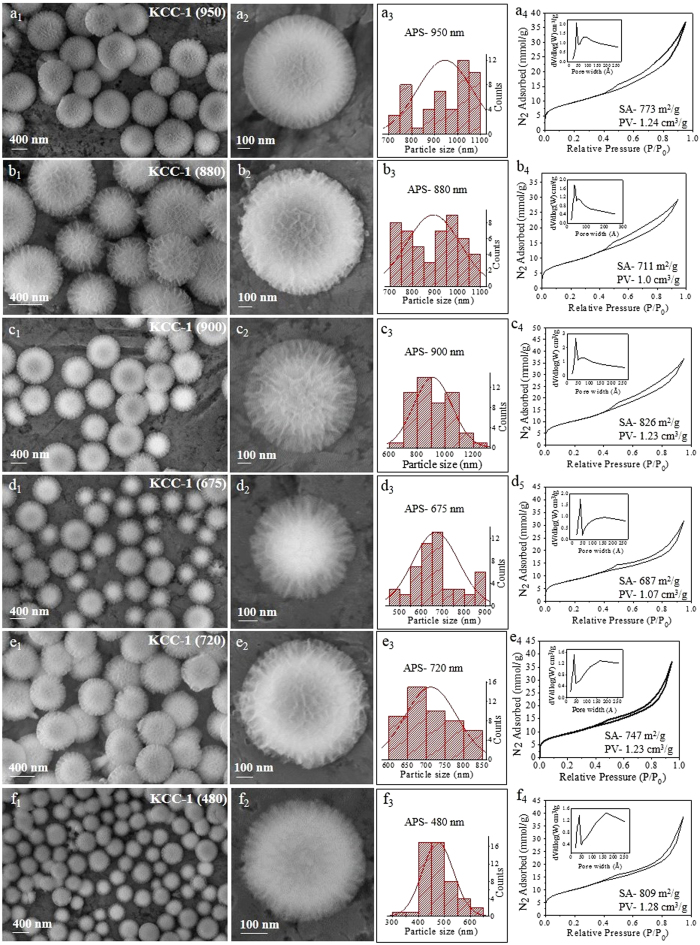
SEM images, particle size distribution, and N_2_ sorption isotherm (pore size distribution in inset) with changes in urea concentration to (**a**) 0.9 g, (**b**) 1.8 g, (**c**) 3.6 g, (**d**) 7.2 g, (**e**) 28.8 g, and (**f**) 57.6 g. *Other synthesis conditions were kept the same as given in* Table S1.

To achieve control over the particle size and fiber density, we first studied the effect of urea concentration. SEM images of the synthesized KCC-1 silica nano-spheres with varying amounts of urea are shown in Fig. 1, indicating the formation of various sized KCC-1 particles with different fiber densities. The average particle size could be varied between 480 and 950 nm, with a BET surface area (SA) from 687 to 826 m^2^/g and a pore volume (PV) from 1.0 to 1.28 cm^3^/g (Fig. 1, Table S1). The change in the initial urea concentration from 0.9 g (15 mmol) to 3.6 g (60 mmol) did not affect the particle size, which was maintained at approximately 900 nm (Fig. 1a–c). However, with a further increase in the urea concentration, a significant effect on the particle size was observed, with 7.2 g yielding 687 nm and 57.6 g yielding 480 nm APS KCC-1 spheres, respectively (Fig. 1d,f). Thus, we observe that with an increase in the urea concentration from 0.9 g to 57.6 g, the APS of the KCC-1 decreases (Fig. 1). We did not observe significant changes in the surface area or pore volume with the change in the urea concentration. The pore size distribution also remained same, with a major pore size of 3.7 nm and a distribution of other larger mesopores (Fig. 1). The N_2_ adsorption-desorption isotherm also showed a similar type IV pattern with a hysteresis loop (Fig. 1a_4_–f_4_).

These results indicate the presence of a nucleation-seed-growth step in the formation mechanism of KCC-1. With the increase in the urea concentration, that acts as a hydrolyzing and condensation reagent, the hydrolysis of the starting precursor TEOS increases, increasing the nucleation and thus the seed formation in the initial stage of the reaction. Because most of the TEOS molecules were already utilized for the nuclei-seed formation, few TEOS molecules were available for the growth of the particles in the growth stage. It is well known that when more seeds are formed in the initial stage, small nanoparticle formation takes place, which we clearly observed in this case also.

**Figure 2:**
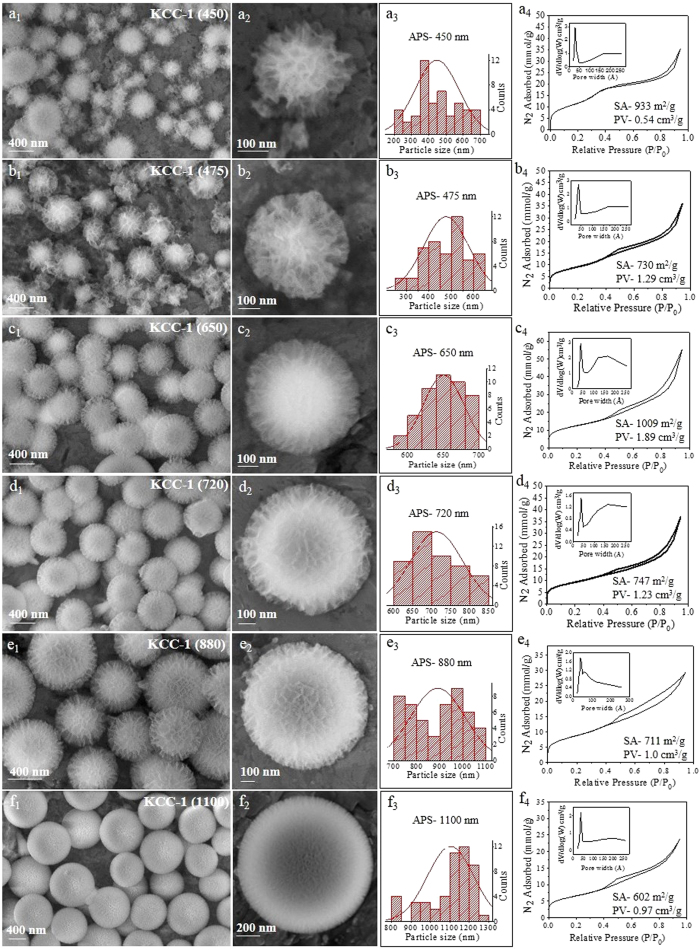
SEM images, particle size distribution, and N_2_ sorption isotherm (pore size distribution in inset) with a urea concentration of 28.8 g (0.44 moles) and reaction hold times of (**a**) 5 min, (**b**) 15 min, (**c**) 30 min, and (**d**) 1 h and with a urea concentration of 1.8 g (0.03 moles) and reaction hold times of (**e**) 1 h and (**f**) 3 h. *Other synthesis conditions were kept the same as given in* Table S1*. KCC-1* (*720*) *is provided here again for easy comparison.*

Based on the above results, we then studied the time-dependent structure evolution of KCC-1. Notably, we observed a step-wise increase in the sphere size with an increase in the reaction hold time (Fig. 2). The time-dependent evolution was studied at two different urea concentrations to achieve the smallest and largest possible sizes of KCC-1 particles. We chose 28.8 g urea for smaller particles and 1.8 g urea to achieve larger particles, as a higher amount of urea yielded smaller particles and a lower amount yielded larger particles (Fig. 1, Table S1). Significantly, with 28.8 g urea, we could tune the particle size from 450 to 720 nm APS. We also observed a gradual increase in the KCC-1 particle size from 450 to 475 to 650 to 720 nm APS with an increase in the reaction hold time from 5 to 15 to 30 to 60 minutes respectively (Fig. 2a–d). The particle size distribution was also narrowed with the increase in reaction time. Using 1.8 g urea, we observed a further increase in the particle size up to 1100 nm (Fig. 2f). Thus, reaction time control allowed the efficient tuning of the particle size to synthesize KCC-1 with different sizes. This also confirms the presence of a growth stage (as discussed in the urea case) in the formation mechanism of KCC-1.

Interestingly, in addition to the changes in size, we also observed variations in the fiber density of the spheres with time at both urea concentrations, and they became denser with the increase in hold time. Although no systematic pattern was observed for the surface area, pore volume and N_2_ sorption isotherm in this case, the pore size distribution became narrower with the increase in fiber density, having 3.7 nm as a major pore, with the mesopore distribution decreasing with the increase in fiber density (Fig. 2a_4_–f_4_). These results indicate that with time, the spheres do not only grow larger, but more fibers are formed, making them denser.

**Figure 3:**
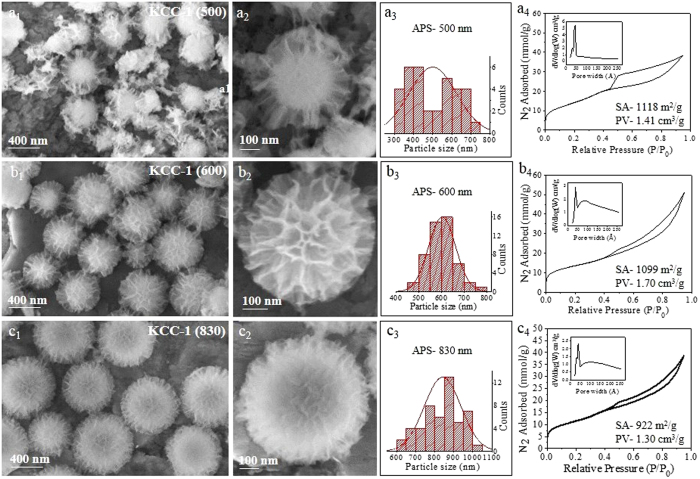
SEM images, particle size distribution, and N_2_ sorption isotherm (pore size distribution in inset) with CTAB concentrations of (**a**) 1.5 g, (**b**) 3 g and (**c**) 6 g. *Other synthesis conditions were kept the same as given in* Table S1.

We also studied the effect of the CTAB concentration on the particle size and textural properties. With the increase in the CTAB concentration, the particle size increased from 500 to 830 nm APS (Fig. 3). Using 1.5 g CTAB, KCC-1 with 500 nm APS was obtained with a high surface area of 1118 m^2^/g and a pore volume of 1.41 cm^3^/g (Fig. 3). Notably, the pore size distribution was narrower, with no larger pores present (Fig. 3a_4_). When the CTAB amount was increased to 3 g, KCC-1 with an APS of 600 nm and less dense fibers was obtained, with a narrow particle size distribution. The pore volume increased to 1.7 cm^3^/g, and there was a similar surface area of 1099 m^2^/g. With a further increase in the CTAB amount to 6 g, the spherical size further increased to 830 nm, with a similar pore volume and surface area. In this case, the fiber density increases, with a narrower pore size distribution. The role of CTAB is not clear yet, but one possibility could be that it might hinder the nucleation/seed formation in the initial stage when present in excess. This would leave more unreacted molecules of TEOS for a longer growth stage, which could increase the particle size.

**Figure 4:**
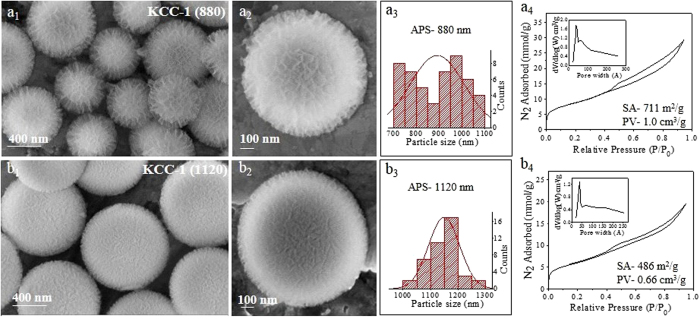
SEM images, particle size distribution, and N_2_ sorption isotherm (pore size distribution in inset) with reaction temperatures of (**a**) 120 °C and (**b**) 140 °C. *Other synthesis conditions were kept the same as given in* Table S1*. KCC-1* (*880*) *is reproduced here for easy comparison.*

Because both the nucleation and growth stages can be affected by the reaction temperature, we studied the effect of the reaction temperature on the size and textural properties of KCC-1 (Fig. 4). We did not observe KCC-1 formation at 100 °C in 1 h reaction time, although very small (20–50 nm) and nearly spherical particles were formed (not shown in the figure). However, at 120 and 140 °C, KCC-1 with an APS of 880 and 1120 nm were formed, respectively (Fig. 4). With the increase in reaction temperature, not only the size increased but also an increase in the fiber density was observed. KCC-1 (1120) was denser than KCC-1 (880), with a surface area reduced to 486 m^2^/g compared to 711 m^2^/g. Though the N_2_ sorption isotherms had similar patterns, the pore volume of KCC-1 (1120) was less 0.66 cm^3^/g compared to the 1.0 cm^3^/g of KCC-1 (880). The pore size distribution also became narrower with an increase in the reaction temperature, with smaller mesopores in KCC-1 (1120) (Fig. 4a4–b4).

**Figure 5:**
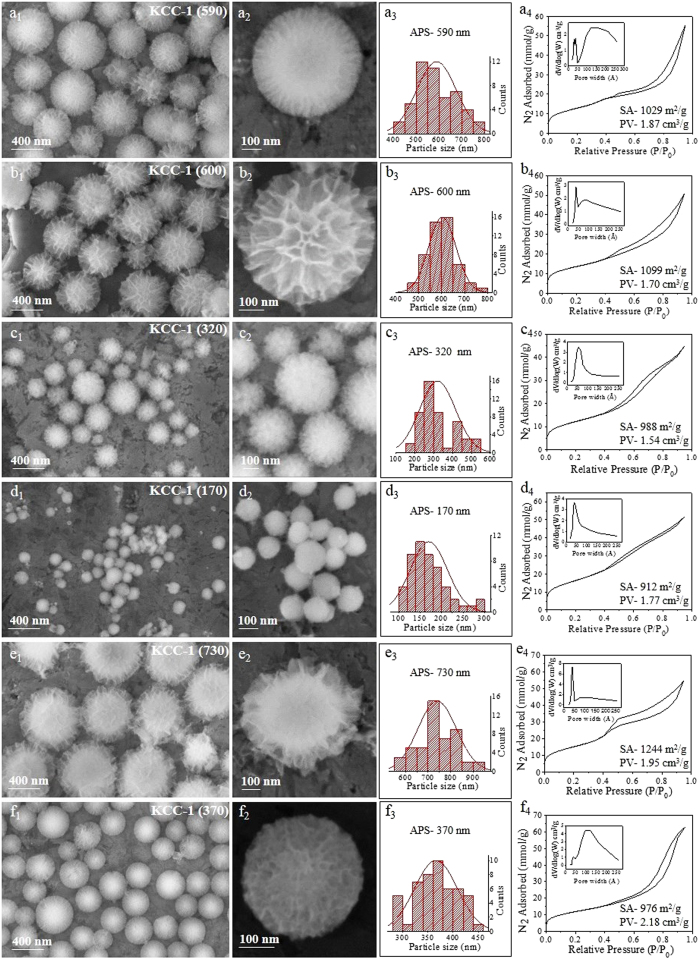
SEM images, particle size distribution, and N_2_ sorption isotherm (pore size distribution in inset) with 1-pentanol concentrations of (**a**) 9 mL, (**b**) 18 mL, (**c**) 36 mL, and (**d**) 72 mL, (**e**) 1-octanol (18 mL), and (**f**) 1-propanol (18 mL). *Other synthesis conditions were kept the same as given in* Table S1*. KCC-1* (*600*) *is reproduced here for easy comparison.*

Although by changing the urea and CTAB amounts, reaction time and temperature we were able to achieve good control over the particle size and fiber density/pore volume, we wanted to achieve even smaller particle sizes to make them useful in bio-related applications such as drug delivery. Therefore we studied the effect of the 1-pentanol concentration and we were amazingly able to synthesize KCC-1 with an APS of 170 nm (Fig. 5d). We observed a gradual decrease in the particle size from approximately 600 to 170 nm APS with an increase in the 1-pentanol concentration from 18 mL to 72 mL (Fig. 5a3–d3). Although the N_2_ sorption isotherm showed a similar pattern with only a slight change in the hysteresis loop, the particle size distribution became narrower with the decrease in the particle size (Fig. 5a4–d4). Notably, KCC-1 (590) showed a very high pore volume of 1.87 cm^3^/g and a pore size distribution ranging from 11.8 to 21.0 nm, in addition to the sharp 3.7 nm pore. This indicates that the increase in pore volume is due to an increase in the number of larger mesopores. KCC-1 (370) showed a sharp pore of approximately 5.5 nm, while KCC-1 (370) showed one of 3.8 nm. Notably, the surface area in all the cases was approximately 1000 m^2^/g. Interestingly, when we replaced 1-pentanol with 1-octanol or 1-propanol, the KCC-1 particle size and textural properties changed dramatically (Fig. 5e,f). 1-Octanol yielded KCC-1 with an APS of 730 nm, while 1-propanol yielded a 370 nm APS. The other textual properties also changed significantly. The pore volume for KCC-1 (730) was 1.95 cm^3^/g, while for KCC-1 (370), it was 2.18 cm^3^/g, the highest reported to date for such materials^17^,^18^,^19^,^20^,^21^,^22^,^23^,^24^,^25^,^26^,^27^,^28^,^29^,^30^,^31^,^32^,^33^,^34^,^35^,^36^,^37^,^38^,^39^,^40^,^41^,^42^,^43^,^44^,^45^,^46^,^47^,^48^,^49^,^50^,^51^,^52^,^53^,^54^,^55^. Its surface area was also very high (1244 m^2^/g), double the previously reported surface area value for KCC-1^17–55^. Even the pore size distribution changed, with 3.8 nm narrow pores for KCC-1 (730) compared to broad 9.5 to 12.1 nm pores for KCC-1 (370). Although the N_2_ sorption isotherm patterns were same, the hysteresis loop started at a lower pressure in the case of KCC-1 (730) compared to KCC-1 (370). This clearly indicates the role of the co-surfactant (1-pentanol) in stabilizing the micelles/microemulsion droplets, thus affecting the size and textural properties. These results indicate the Winsor-type system^28^ or microemulsion-type^29^ mechanism responsible for KCC-1 formation, rather than a micelle-assisted seed-growth mechanism.

**Figure 6:**
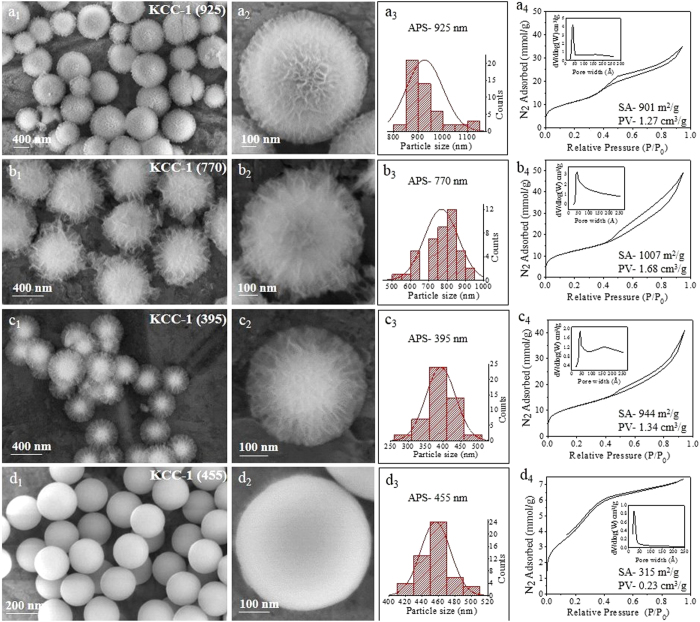
SEM images, particle size distribution, and N_2_ sorption isotherm (pore size distribution in inset) with solvent ratios of water: cyclohexane of (**a**) 450:150 mL, (**b**) 150:450 mL, (**c**) 15:600 mL, and (**d**) 600:0 mL *Other synthesis conditions were kept same as given in* Table S1*. In c, 15 mL of water was needed to dissolve the urea.*

Because we are using two immiscible solvents, water and cyclohexane, their ratio might also affect the particle size and other properties. To obtain insight on this effect, we synthesized KCC-1 in different water: cyclohexane solvent ratios, i.e., 450:150 mL, 150:450 mL, 15:600 mL (15 mL water to dissolve urea) and 600:0 mL (Fig. 6). As expected, when only water was used as a solvent, no KCC-1 fibrous spheres were formed, and solid Stober-like silica spheres were formed, with a reduced surface area and pore volume of 315 m^2^/g and 0.23 cm^3^/g, respectively, indicating its non-porous structure (Fig. 6d). Interestingly, when the cyclohexane amount was increased, the particle size was reduced from 925 nm APS to 395 nm APS. All of them possessed a good surface area and pore volume (Fig. 6a4–c4). Thus, one can also tune the particle size by tuning the solvent ratio.

**Figure 7:**
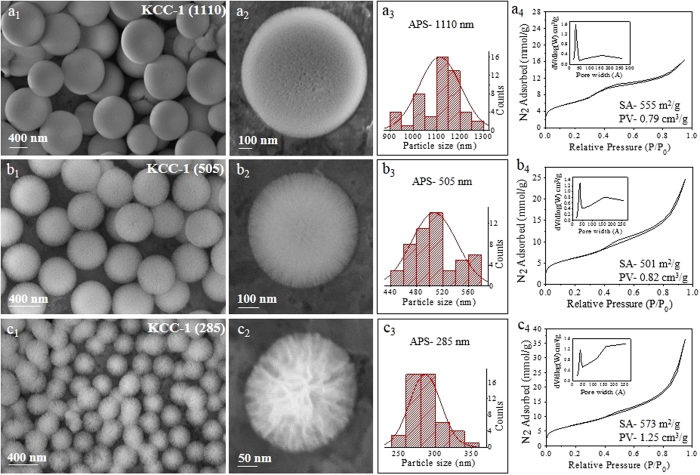
SEM images, particle size distribution, and N_2_ sorption isotherm (pore size distribution in inset) with outside stirring times (time between 1-pentanol addition and microwave exposure) of (**a**) 30 min, (**b**) 2 h, and (**c**) 5 h. *Other synthesis conditions were kept the same as given in* Table S1.

Inadvertently, we also observed the effect of the outside stirring time on the KCC-1 size. Surprisingly, with an increase in the outside stirring time (at room temperature, before exposure to microwaves), we observed a drastic change in the particle size (Fig. 7). 30 minutes of outside stirring yielded an 1110 nm APS, while a 2 h stirring time reduces the APS to 505 nm, and, more fascinatingly, a 5 h stirring time reduces the particle size further to 285 nm APS (Fig. 7). The particle size distribution also became narrower with an increase in the outside stirring time. Other properties did not change drastically. These results indicate that the nucleation-seed stage might have started at room temperature even before exposure to microwave/heating. A longer stirring time allowed for greater seed formation and hence a smaller final particle size. On the other hand, if the microemulsion droplet formation mechanism is to be considered true, these droplets (which acts as reactor for KCC-1 formation) might get smaller with the stirring time, reducing the particle size of KCC-1 for that reason.

We also attempted to obtain detailed insight into the formation mechanism of KCC-1. Previously, we proposed a reverse micellar mechanism^17^, and others proposed a Winsor system or microemulsion-based mechanism for its formation^28^,^29^. We tested several techniques to monitor this micelle or microemulsion droplet formation, such as cryo-TEM, cryo-SEM, dynamic light scattering (DLS), and high-resolution optical microscopy. Unfortunately, we were unable to obtain any meaningful data, mainly due to the small size and instability of the micelles or microemulsion droplets. However, based on our observations in this study and a report by Ganguly *et al.*^57^, we feel that the reverse micellar mechanism or microemulsion based on these reverse micelles may be probable mechanism. First, a seed is formed using a reverse micelle as a template, and then growth takes place without any template. This explains the formation of larger-size particles using few-nm micelles. However, based on the results obtained by changing the solvent ratio and 1-pentanol concentration, the microemulsion droplet formation as well as the Winsor II mechanism seems correct. At this stage, we are not sure about the formation mechanism and currently making strong attempts to study it using other techniques.

## Conclusions

We are able to synthesize KCC-1 with various particles size, surface area, pore volume and fiber density. Now, one can synthesize KCC-1 with a particle size as small as 170 nm to as large as 1120 nm. We are also able to double the surface area of KCC-1 to 1244 m^2^/g and achieve a pore volume of 2.18 cm^3^/g, which are the highest values reported to date for KCC-1.

Smaller particles can be used for various biological applications such as drug delivery, while lager particles can be used for catalysis and photocatalysis, with improved stability due to the larger particle size and improved light harvesting due to the greater internal scattering. Notably, one can now synthesize KCC-1 having a similar size but different surface area, pore volume and fiber density. This will allow a systematic study on the effect of each of these parameters on catalysis, adsorption, light harvesting and various other applications.

We were also able to tune the fiber density and pore volume. Notably, we observed a direct relationship between the fiber density and pore volume. With increases in the fiber density, the pore volume decreases, as the space between the fibers that provides the overall volume is reduced. We also observed the relationship between the fiber density and pore size distribution. The pore size distribution became narrower with an increase in fiber density, due to the decrease in the large mesopore region. This may be due to the V-shape pore/fiber channel in KCC-1, which will have pore sizes ranging from a few nm (3.7 nm, as the major pore may be close to the point of origin/center point of the sphere) and gradually increasing in width due to the V-shape structure. Now, when the fiber density is lower, the fibers are relatively distant from each other, and the V-shape is broadened, which increases the density of the larger pores and reduces the number of small pores. On the other hand, an increase in the fiber density causes their straightening (contraction in V-shape), and thus the majority of pores were approximately 3.7 nm and few larger pores were observed. A relationship was also established between the fiber density and the surface area and pore volume. Spheres with less dense fibers have more surface area/pore volume, and dense spheres have less surface area/pore volume.

Thus, in this work, we have shown that the particle size, fiber density, surface area, and pore volume of KCC-1 can be efficiently tuned by changing various reaction parameters, such as the urea concentration, CTAB concentration, reaction time and temperature, concentration of 1-pentanol, solvent ratio, and even outside stirring time. This customization/tailoring ability of KCC-1 spheres will broaden as well as open up applications in many other fields, and we strongly feel that more researchers will now use KCC-1 over popular MCM-41 and SBA-15 silicas.

## Methods

### Procedure for synthesis of size and fiber density controlled KCC-1

In a typical procedure, cetyltrimethylammonium bromide (CTAB) (3 gm, 8.23 mmol) and urea (3.6 gm, 60 mmol) were first dissolved in deionized water (300 mL) by vigorously stirring at 1400 rpm for 10 minutes in a 2 L conical flask. To the above mixture, a solution of tetraethoxysilane (TEOS) (72 mmol) in cyclohexane (300 mL) was added dropwise over 20 minutes under stirring by using a dropping funnel and then stirred at 1400 rpm for 10 minutes. 1-Pentanol (18 mL) was then dropwise added to above mixture in 5 minutes under stirring, and the mixture was further stirred for several minutes (outside stirring time). The reaction mixture was then transferred into a 1 L microwave reactor and exposed to microwave radiation (maximum power- 800 W) to achieve the required temperature via a 30-minute ramp from room temperature (23 °C), with 50% stirring speed in the Ethos-1 microwave reactor. At each temperature, the reaction mixture was maintained (hold time) for a few minutes to several hours (Table S1) and then allowed to cool to room temperature naturally. The solid product was isolated by centrifugation and washed with ethanol (3 times) and water (3 times), followed by air drying. The product was calcined at 550 °C for 6 h in air to yield pure KCC-1. The exact values of the chemicals/solvents used, reaction temperature, reaction time, and outside stirring time are provided in Table S1 and in the caption of each figure. All the materials were characterized by scanning electron microscopy (SEM), transmission electron microscopy (TEM), low and wide angle powder X-ray diffraction and N_2_ sorption analysis.

## Additional Information

**How to cite this article**: Bayal, N. *et al.* Size and Fiber Density Controlled Synthesis of Fibrous Nanosilica Spheres (KCC-1). *Sci. Rep.* **6**, 24888; doi: 10.1038/srep24888 (2016).

## Supplementary Material

Supplementary Information
