# Supplementary material for: Size and Fiber Density Controlled Synthesis of Fibrous Nanosilica Spheres (KCC-1)
Source: Sci Rep. 2016 Apr 27;6:24888. doi: 10.1038/srep24888 (PMC4846819; doi:10.1038/srep24888)
Supplement: Supplementary Information [file srep24888-s1.pdf]

Size and Fiber Density Controlled Synthesis of Fibrous Nanosilica Spheres (KCC-1)

Nisha Bayal,[#] Baljeet Singh,[#] Rustam Singh and Vivek Polshettiwar *

Nanocatalysis Laboratories (NanoCat), Department of Chemical Sciences, Tata Institute of Fundamental Research (TIFR), Mumbai, India, Email: vivekpol@tifr.res.in,

# - Equal contribution

Table 1: Summary of various as-synthesised KCC-1, by changing various reaction parameters.

Sample Name	TEOS (gm)	CTAB (gm)	Urea (gm)	Water (mL)	Cyclohexane (mL)	Pentanol (mL)	Outside Stirring Time (min)	Reaction Temp. (^{0}C)	Hold Time (min)	Average Particle Size (APS) nm	BET Surface Area cm^2/gm	Pore Volume cm^3/gm
**Effect of Urea**												
KCC-1 (950)	15	3	**0.9**	300	300	18	20	120	60	950	773	1.24
KCC-1 (880)	15	3	**1.8**	300	300	18	20	120	60	880	711	1.0
KCC-1 (900)	15	3	**3.6**	300	300	18	20	120	60	900	826	1.23
KCC-1 (675)	15	3	**7.2**	300	300	18	20	120	60	675	687	1.07
KCC-1 (720)	15	3	**28.8**	300	300	18	20	120	60	720	747	1.23
KCC-1 (480)	15	3	**57.6**	300	300	18	20	120	60	480	809	1.28
**Effect of Time**												
KCC-1 (450)	15	3	28.8	300	300	18	20	120	**5**	450	933	0.54

KCC-1 (475)	15	3	28.8	300	300	18	20	120	**15**	475	730	1.29
KCC-1 (650)	15	3	28.8	300	300	18	20	120	**30**	650	1009	1.89
KCC-1 (720)	15	3	28.8	300	300	18	20	120	**60**	720	747	1.23
KCC-1 (880)	15	3	1.8	300	300	18	20	120	**60**	880	711	1.0
KCC-1 (1100)	15	3	1.8	300	300	18	20	120	**180**	1100	602	0.97
**Effect of CTAB**												
KCC-1 (500)	15	**1.5**	3.6	300	300	18	20	120	30	500	1118	1.41
KCC-1 (600)	15	**3**	3.6	300	300	18	20	120	30	600	1099	1.70
KCC-1 (830)	15	**6**	3.6	300	300	18	20	120	30	830	922	1.30
**Effect of Temperature**												
KCC-1 (880)	15	3	1.8	300	300	18	20	**120**	60	880	711	1.0
KCC-1 (1120)	15	3	1.8	300	300	18	20	**140**	60	1120	486	0.66
**Effect of 1-pentanol**												
KCC-1 (590)	15	3	3.6	300	300	**9**	20	120	30	590	1029	1.87
KCC-1 (600)	15	3	3.6	300	300	**18**	20	120	30	600	1099	1.7
KCC-1 (320)	15	3	3.6	300	300	**36**	20	120	30	320	988	1.54
KCC-1 (170)	15	3	3.6	300	300	**72**	20	120	30	170	912	1.77
KCC-1 (730)	15	3	3.6	300	300	**18 (1-Octanol)**	20	120	30	730	1244	1.95
KCC-1 (370)	15	3	3.6	300	300	**18 (1-Propanol)**	20	120	30	370	976	2.18
**Effect of Solvent ratios**												
KCC-1 (925)	15	3	3.6	**450**	**150**	18	20	120	30	925	901	1.27

KCC-1 (770)	15	3	3.6	**150**	**450**	18	20	120	30	770	1007	1.68
KCC-1 (395)	15	3	3.6	**15**	**600**	18	20	120	30	395	944	1.34
KCC-1 (455)	15	3	3.6	**600**	**0**	18	20	120	30	455	315	0.23
**Effect of outside stirring time**												
KCC-1 (1110)	30	3	1.8	300	300	18	**30**	120	180	1110	555	0.79
KCC-1 (501)	30	3	1.8	300	300	18	**120**	120	180	505	501	0.82
KCC-1 (573)	30	3	1.8	300	300	18	**300**	120	180	285	573	1.25
